# Tuning Dielectric Loss of SiO_2_@CNTs for Electromagnetic Wave Absorption

**DOI:** 10.3390/nano11102636

**Published:** 2021-10-07

**Authors:** Fenghui Cao, Jia Xu, Xinci Zhang, Bei Li, Xiao Zhang, Qiuyun Ouyang, Xitian Zhang, Yujin Chen

**Affiliations:** 1 Key Laboratory of In-Fiber Integrated Optics, College of Physics and Optoelectronic Engineering, Harbin Engineering University, Harbin 150001, China; caofenghui@hrbeu.edu.cn (F.C.); xujia110006@hrbeu.edu.cn (J.X.); zhangxinci@hrbeu.edu.cn (X.Z.); 1284034781@hrbeu.edu.cn (B.L.); qyouyang7823@aliyun.com (Q.O.); 2 School of Mechatronic Engineering, Daqing Normal University, Daqing 163712, China; 3 Key Laboratory for Photonic and Electronic Bandgap Materials, Ministry of Education, School of Physics and Electronic Engineering, Harbin Normal University, Harbin 150025, China; xtzhangzhang@hotmail.com; 4 School of Materials Science and Engineering, Zhengzhou University, Zhengzhou 450001, China

**Keywords:** CNTs, dielectric loss, nitrogen doping, electromagnetic wave absorption

## Abstract

We developed a simple method to fabricate SiO_2_-sphere-supported N-doped CNTs (NCNTs) for electromagnetic wave (EMW) absorption. EMW absorption was tuned by adsorption of the organic agent on the precursor of the catalysts. The experimental results show that the conductivity loss and polarization loss of the sample are improved. Meanwhile, the impedance matching characteristics can also be adjusted. When the matching thickness was only 1.5 mm, the optimal 3D structure shows excellent EMW absorption performance, which is better than most magnetic carbon matrix composites. Our current approach opens up an effective way to develop low-cost, high-performance EMW absorbers.

## 1. Introduction

With the development of science and technology, the rapid rise of artificial intelligence, the popularity of the smart home, and the extensive application of various electrical and electronic products, people’s work efficiency and quality of life has improved. However, at the same time, the widespread use of electronic products also hides huge harms: long-term exposure to electromagnetic radiation will damage human health, but also harms other electronic products’ electromagnetic interference, affecting their normal work. These hazards have attracted the attention of many countries in the world, and development of efficient electromagnetic absorption and shielding materials has become the main research direction. Therefore, it is necessary to develop a high-performance electromagnetic wave (EMW) absorber. To improve the efficiency of the unitizations, lightweight absorbers with a thin thickness are required. Carbonaceous materials such as graphene, carbon nanotubes (CNTs) and carbon nanofibers have attracted great attention because of their low mass density, good mechanical and chemical stability and high surface areas [1,2,3,4,5,6,7,8,9]. Carbon nanotubes have received extensive attention and in-depth studies in the field of EMW absorption due to their tubular structure suitable for electron transport, their light weight and good electrical conductivity [10,11,12,13,14,15,16,17]. For example, Lv et al. encapsulated Fe/Fe_3_C nanoparticles (NPs) into N-doped CNTs (NCNTs) and obtained the result that the sample had a reflection loss (*R*_L_) of −46.0 dB and a thickness of 4.97 mm at 3.6 GHz [10]. Chang et al. reported Fe_3_O_4_/PPy/CNT composites with an *R*_L_ of −25.9 dB with a thickness of 3.0 mm [11]. The reflection loss of ZnFe_2_O_4_@CNT/PVDF composite film prepared by Li et al. was −54.5 dB, with a matching thickness of 2.4 mm [12]. Gong et al. reported SiCN(Fe) fibers with an *R*_L_ of −47.64 dB and the effective absorption bandwidth of 4.28 GHz [13]. The minimum reflection loss of Fe_3_O_4_/CNTs prepared by Zeng et al. was −51.0 dB, with a matching thickness of 4.4 mm [14]. Recently, a series of magnetic metal alloys (Fe, Co, Ni, etc.) that encapsulated into NCNTs were designed for EMW absorption [15,16]. However, the impedance matching feature of the composites mentioned above needed to be precisely tuned due to highly conductive magnetic metals and CNTs in these composites [17]. Furthermore, there is still room to improve the EMW absorption property of CNT-based absorbers, such as stronger absorption at a lower filler ratio and thinner matching thickness.

Here, we propose a simple method for SiO_2_-sphere-supported NCNTs with embedded Fe_3_C/Fe nanoparticles (NPs) (SiO_2_@Fe_3_C/Fe@NCNT-GT) for EMW absorption. Fe(OH)_X_ was first coated on the surface of SiO_2_ spheres [18], and then the organic solvent (terephthalic acid) was adsorbed on the Fe(OH)_x_ surface to form relatively larger metal NPs for the growth of NCNTs with moderate diameters. Compared to the counterpart (SiO_2_@Fe_3_C/Fe@NCNT) without treatment in the organic solvent, the as-prepared SiO_2_@Fe_3_C/Fe@NCNT-GT showed significantly improved EMW absorption performance. At a filler ratio of 25%, the minimum *R*_L_ (*R*_L, m__in_) and effective bandwidth of the SiO_2_@Fe_3_C/Fe@NCNT-GT reached −48.43 dB and 4.51 GHz, respectively, while the matching thickness was only 1.5 mm.

## 2. Materials and Methods

### 2.1. Materials

Tetraethoxysilane (TEOS, 99 wt%, analytical reagent, A.R.) was purchased from Tianjin Komiou Chemical Reagent Co., Ltd. (Tianjin, China). NH_4_OH (25 wt%, A.R.) was purchased from XiLong Scientific Co., Ltd. (Shantou, China). Absolute ethanol (99 wt%, analytical reagent, A.R.) was purchased from Tianjin Fuyu Fine Chemical Co., Ltd. (Tianjin, China). Terephthalic, N,N-Dimethylformamide (DMF) and dicyandiamide were purchased from Tianjin Guangfu Fine Chemical Research Institute (Tianjin, China). Ferric acetylacetonate was purchased from Sinopharm Chemical Reagent Co., Ltd. (Shanghai, China). Paraffin was purchased from Yuyang Wax Industry (Changge, China). It is worth noting that all chemicals were purchased without further treatment before use, and all aqueous solutions were prepared using ultrapure water.

### 2.2. Characterizations and Electromagnetic Parameter Measurement

The morphology and size of the samples were characterized using scanning electron microscopy (SEM; Hitachi SU70, Tokyo, Japan) and transmission electron microscopy (TEM; FEITecnai-F20, Hillsboro, USA). Energy dispersive X-ray spectroscopy (EDX) was performed to confirm the elemental contents of the samples. X-ray diffraction (XRD) data were measured using a Rigaku D/max 2550 V (Tokyo, Japan) with Cu Kα radiation (λ = 1.5418 Å). X-ray photoelectron spectroscopy (XPS) analyses were carried out by using a spectrometer with Mg Kα radiation (ESCALAB 250, Shanghai, China). Raman spectra were recorded on a Raman spectrometer (Xplora Plus, Paris, France) using a 488 nm He−Ne laser. The Brunauer–Emmett–Teller (BET) surface area and pore volume were tested with a Quantachrome Instruments Autosorb-iQ2-MP (Beijing, China) after the composites were vacuum dried at 200 ℃ for 10 h. Fourier-transform infrared (FTIR) spectra of samples were collected using a Nicolet FTIR510 spectrometer (KBr pellet method, 4 cm^−1^ resolution, Waltham, MA, USA). The electromagnetic wave absorption properties of the absorbing materials were measured using a vector network analyzer (Anritsu MS4644A Vectorstar, Kanagawa, Japan) in the 2–18 GHz range at room temperature.

### 2.3. Methods

#### 2.3.1. Synthesis of the SiO_2_

Synthesis of the SiO_2_ was conducted following the Stöber method. Deionized water (18 mL), absolute ethanol (76 mL) and TEOS (14 mL) were dissolved into NH_4_OH (98 mL), and continuously stirred for 4 h. Then, the colloidal solution was centrifuged, and the resultant was placed in an oven at 100 °C for 12 h to obtain silica microspheres [19,20].

#### 2.3.2. Synthesis of the SiO_2_@Fe(OH)_x_

SiO_2_ with diameters of about 400 nm (216 mg) were first dispersed in ethanol (72 mL), and ferric acetylacetonate (270.5 mg) was added to the mixture above, sequentially. Distilled water (3.6 mL) and ammonia (2 mL) were added to the mixture with sonication for 15 min. The as-prepared mixture was sealed in a conical flask and stirred at 80℃ for 10 h. The precipitate was washed with distilled water and ethanol several times, then centrifuged and dried in a vacuum oven at 40 °C to obtain the SiO_2_@Fe(OH)_x_ [20].

#### 2.3.3. Synthesis of the SiO_2_@Fe_3_C/Fe@NCNT

The SiO_2_@Fe(OH)_x_ was annealed in Ar (the temperature was 800 °C, the time was 30 min and the ramp rate was 5 °C/min) to obtain SiO_2_@Fe_3_C/Fe@NCNT [20].

#### 2.3.4. Synthesis of the SiO_2_@Fe(OH)_x_-GT

The SiO_2_@Fe(OH)_x_ (200 mg), terephthalic acid (300 mg), pure water (3 mL) and ethanol (3 mL) were added into N,N-Dimethylformamide (DMF) solution (30 mL), and stirred for 30 min. Then, the mixed solution was placed into a 50 mL Teflon container and was treated at a high temperature of 150 °C for 12 h. The precipitate was washed and dried in a 40 °C vacuum oven to obtain the SiO_2_@Fe(OH)_x_-GT.

#### 2.3.5. Synthesis of the SiO_2_@Fe_3_C/Fe@NCNT-GT

The SiO_2_@Fe(OH)_x_-GT was annealed in Ar (the temperature was 800 °C, the time was 30 min, and the ramp rate was 5 °C/min) to obtain the SiO_2_@Fe_3_C/Fe@NCNT-GT. The detailed experimental material, structural characterizations and the method are described in the Supplementary Materials.

## 3. Results and Discussion

**Figure 1:**
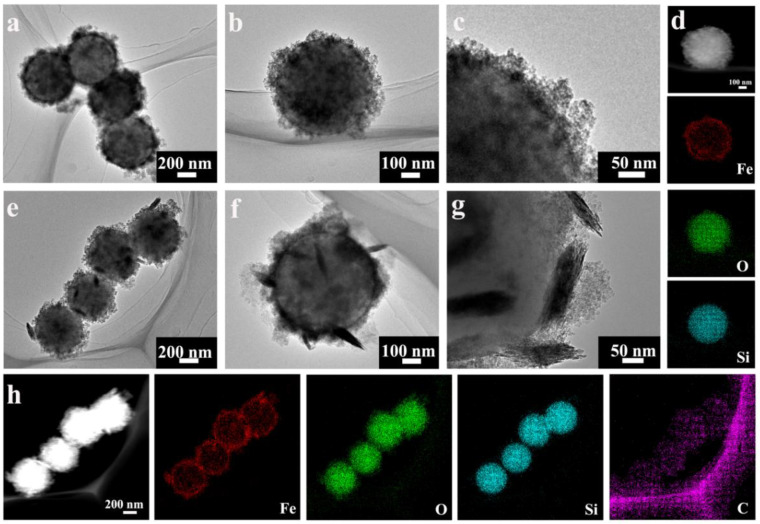
(**a**–**c**) TEM images of structural characterizations and (**d**) EDX element mappings of the SiO_2_@Fe(OH)_x_. (**e**–**g**) TEM image of structural characterizations and (**h**) EDX element mappings of SiO_2_@Fe(OH)_x_-GT.

The diameter of the prepared SiO_2_ microspheres were approximately 400 nm. After being coated with an Fe(OH)_x_ layer on the surface of the SiO_2_ spheres, the diameter increased from 400 nm to about 500 nm (SiO_2_@Fe(OH)_x_). Scanning electron microscopy (SEM) imaging and transmission electron microscopy (TEM) imaging showed that the Fe(OH)_x_ layer was uniformly coated on the surface of the SiO_2_ spheres (Figure 1a–c and Figure S1a, Supplementary Materials). In the X-ray diffraction (XRD) pattern, there were two obvious peaks at 2*θ* 35.02° and 62.72°, indicating that the outmost layer of the SiO_2_@Fe(OH)_x_ was mainly composed of weakly crystalline Fe(OH)_3_, which corresponded to JCPDS card no. 22-0346 (Figure S2a). Energy dispersive X-ray spectroscopy (EDX) element mappings were also confirmed. As shown in Figure 1d, there were Si and O signals in the spherical core region, suggesting that the spherical core region materials were still SiO_2_ spheres. Fe and O single elements were obviously present in the area outside the sphere, confirming the composition of the SiO_2_@Fe(OH)_3_. The XRD pattern of the SiO_2_@Fe(OH)_x_-GT indicated that the weakly crystalline Fe(OH)_3_ (Figure S2a) remained after the treatment, but the rough surface became relatively smooth (Figure S1b). TEM images show that there was a lamellar structure on the surface of the SiO_2_@Fe(OH)_3_-GT, different from the SiO_2_@Fe(OH)_3_ (Figure 1e–g). The peak at 798 and 1431 cm^−1^ in the Fourier-transform infrared (FTIR) spectra of the SiO_2_@Fe(OH)_3_-GT corresponded to the C–H deformation vibration and the C=C stretching vibration (Figure S2b). The peak 1659 cm^−1^ in the spectrum of the SiO_2_@Fe(OH)_3_-GT corresponded to the C=O stretching vibration (Figure S2b). Thus, the FTIR results indicated the adsorption of terephthalic acid on the Fe(OH)_3_ layer. EDX element mappings also confirmed that Fe, O and C single elements were present in the area outside of the sphere, confirming the adsorption of terephthalic acid on the Fe(OH)_3_ layer (Figure 1h).

**Figure 2:**
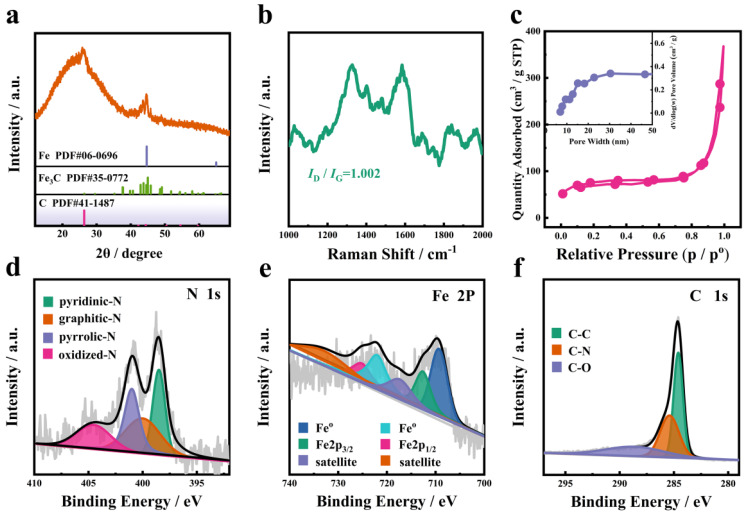
(**a**) XRD pattern, (**b**) Raman spectra, (**c**) pore size distribution and N_2_-sorption isotherm, (**d**–**f**) N 1s, Fe 2p and C 1s XPS spectra of the SiO_2_@Fe_3_C/Fe@NCNT-GT.

In order to analyze the composition and valence state of the SiO_2_@Fe_3_C/Fe@NCNT-GT, XRD and Raman spectra were performed. As shown in Figure 2a, there was a broad diffraction (2*θ)* from 10° to 30°, corresponding to amorphous SiO_2_ in the XRD pattern of the SiO_2_@Fe_3_C/Fe@NCNT-GT. In the XRD pattern of the SiO_2_@Fe_3_C/Fe@NCNT-GT, the peak at 2*θ* 44.7° and 42.9° can be indexed to (110) planes of the Fe NPs (JCPDS no. 06-0696) and (211) planes of the Fe_3_C (JCPDS no. 35-0772), in sequence, while the peak at 26.4° is attributed to the NCNTs (JCPDS no. 41-1487). The Raman spectra of the SiO_2_@Fe_3_C/Fe@NCNT-GT showed two distinguishable peaks: one at 1325 cm^−1^ (D band) and the other at 1585 cm^−1^ (G band) (Figure 2b). Their intensity ratios (*I*_D_/*I*_G_) for the SiO_2_@Fe_3_C/Fe@NCNT-GT was 1.002, which indicated rich defects in the sample. These defects in the SiO_2_@Fe_3_C/Fe@NCNT-GT can greatly improve the polarization relaxation and contribute to enhancing the absorption of electromagnetic waves [21]. As shown in Figure 2c, the N_2_-sorption isotherms of the SiO_2_@Fe_3_C/Fe@NCNT-GT displays type-IV loops, revealing that the mesopores existed in the prepared sample. Furthermore, the Brunauer–Emmett–Teller (BET) surface area of the SiO_2_@Fe_3_C/Fe@NCNT-GT was 243.54 m^2^ g^−1^. The illustration in Figure 2c displays pore size distribution diagrams. The pore sizes of the SiO_2_@Fe_3_C/Fe@NCNT-GT were centered at 15 nm, and pore volume was 0.568 cm³ g^−1^. X-ray photoelectron spectroscopy (XPS) spectra displayed that there were five elements (Fe, N, O, Si and C) in the SiO_2_@Fe_3_C/Fe@NCNT-GT (Figure S3). The peaks were at 398.5 (pyridine-N), 399.9 (pyrrolic-N), 401.1 (graphite-Nand) and 404.5 eV (oxide-N) in the XPS spectra of N 1s, respectively (Figure 2d) [22]. The peaks at 709.1 and 721.9 eV in the XPS spectra of Fe 2p can be indexed to metallic Fe. The peaks (717.3 and 733.2 eV) and satellite peaks (712.1 and 724.9 eV) reveal the oxidation state of Fe species in the sample [23,24,25] (Figure 2e). The binding energies of C–C (284.6 eV), C–N (285.7 eV) and C–O (288.7 eV) were observed on the surface of NCNT (Figure 2f) [26,27]. The carbon atom will tend to form unsaturated covalent bonds with the oxygen anion, increasing the charge state and increasing the band gap. This is due to the electron density shifts from the carbocation to the more electronegative oxygen anion, which in turn affects the electron structure.

**Figure 3:**
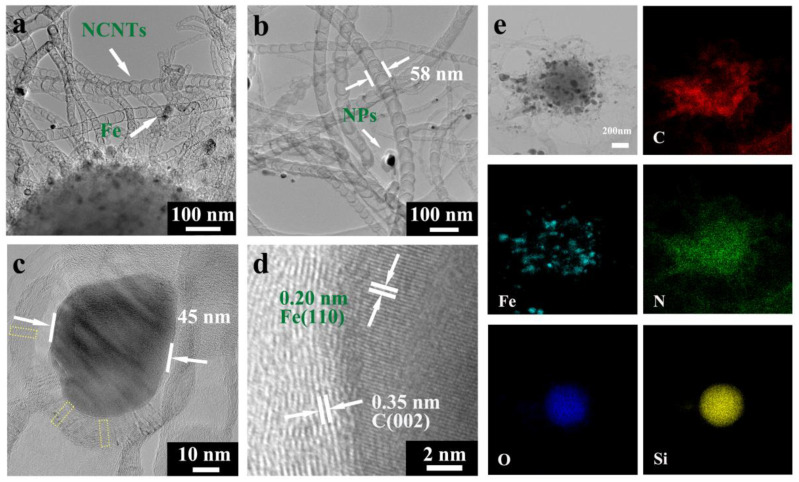
(**a**–**c**) TEM images, (**d**) HRTEM images, (**e**) TEM image and EDX elemental mappings of SiO_2_@Fe_3_C/Fe@NCNT-GT. The defects are marked by yellow dotted square (**c**).

SEM images indicated that the SiO_2_@Fe_3_C/Fe@NCNT-GT exhibited 3D morphology, where the NCNTs were grown on the surface of the SiO_2_ spheres (Figure S4). Bamboo-like NCNTs in the SiO_2_@Fe_3_C/Fe@NCNT-GT are also observed in Figure 3a,b with a length of approximately 1.5 μm. Magnified TEM images show that their average diameter and wall thickness were approximately 51 and 12 nm, respectively (Figure 3b,c and Figure S5). There were some NPs, with an average diameter of about 39 nm, embedded in the bamboo-like NCNTs. Figure 3c shows that the NPs were encapsulated in 25–30 layers of the graphene shell in the high-resolution TEM (HRTEM) images. The *d*-spacing of labeled lattice fringes of 0.20 nm corresponded to the (110) planes of Fe, while the *d*-spacing of 0.35 nm corresponded to the (002) planes of graphite–carbon (Figure 3d). Notably, a mass of defects were present in the NCNT walls and graphene shell of the SiO_2_@Fe_3_C/Fe@NCNT-GT. As is shown in Figure 3c, these defects are marked with a yellow frame. Defects including lattice distortion, lattice dislocation and fracture edges are considered to have a positive effect on the absorption property of the SiO_2_@Fe_3_C/Fe@NCNT-GT. The distribution of elements in the SiO_2_@Fe_3_C/Fe@NCNT-GT was analyzed by EDX element mapping. There were O and Si signals in the spherical core zone, indicating that the spherical core region mediums were still SiO_2_ spheres (Figure 3e). Fe, C and N single elements were present in the zone of the NCNTs, confirming the composition of NCNTs. Compared to the SiO_2_@Fe_3_C/Fe@NCNT [20], the average diameter of the NCNTs in the SiO_2_@Fe_3_C/Fe@NCNT-GT increased from 15 nm to 58 nm, the length of bamboo nodes increased from 15 nm to 50 nm, and the wall thickness increased from 3 to 12 nm. The adsorption of terephthalic acid limited the contact of the Fe(OH)_3_ with the reductive gases, leading to the formation of the larger metal NPs. Consequently, the diameter of NCNTs became larger compared to the counterpart without the adsorption of terephthalic acid.

**Figure 4:**
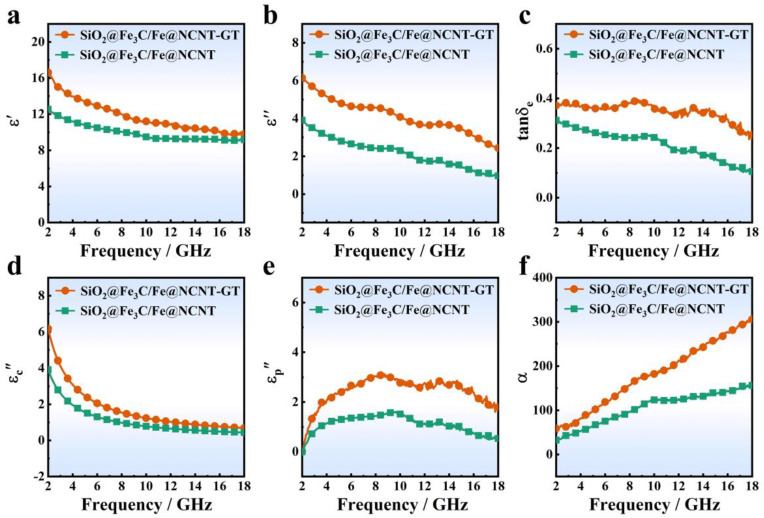
(**a**) *ε*′—*f* curves, (**b**) *ε″*—*f* curves, (**c**) tan*δ*_e_—*f* curves, (**d**) *ε_c_″*—*f* curves, (**e**) *ε_p_″*—*f* curves and (**f**) α—*f* curves of SiO_2_@Fe_3_C/Fe@NCNT-GT and SiO_2_@Fe_3_C/Fe@NCNT.

The factors that may enhance the absorption performance of EMW were investigated through the comparison of electromagnetic parameters of the SiO_2_@Fe_3_C/Fe@NCNT-GT and the SiO_2_@Fe_3_C/Fe@NCNT, including complex permittivity and permeability. They can be expressed separately by the formula *ε*_r_ = *ε′* − *jε″* and *μ*_r_ = *μ′* − *jμ″*. (*ε′* is the real part of permittivity, *ε″* is the imaginary part of permittivity, *μ′* is the real part of permeability, and *μ″* is the imaginary part of permeability) [28,29,30]. As shown in Figure 4a–c, the *ε′* values of the SiO_2_@Fe_3_C/Fe@NCNT-GT varied in a range of 16.63−9.81, and the *ε″* values of the SiO_2_@Fe_3_C/Fe@NCNT-GT varied in a range of 6.15−2.44. The permittivity for both samples gradually decreased with the increase in the frequency, which is due to the frequency dispersion effect. The *ε′* and *ε″* values of the SiO_2_@Fe_3_C/Fe@NCNT-GT were larger than those of the SiO_2_@Fe_3_C/Fe@NCNT. The dielectric loss tangent (tan*δ*_e_ = *ε″/ε′*) of the SiO_2_@Fe_3_C/Fe@NCNT-GT was also larger. Figure S6 shows that the two samples had very little difference in the real part of permeability, imaginary part of permeability and magnetic loss tangent (tan *δ*_m_), with a value over 2–18 GHz. The saturation magnetization (*M*_s_), remnant magnetization (*M*_r_) and coercivity (*H*_c_) of the SiO_2_@Fe_3_C/Fe@NCNT-GT were slightly larger than those of the SiO_2_@Fe_3_C/Fe@NCNT (Figure S7). Thus, the magnetic loss of the SiO_2_@Fe_3_C/Fe@NCNT-GT is not a determining factor for the EMW performance.

**Figure 5:**
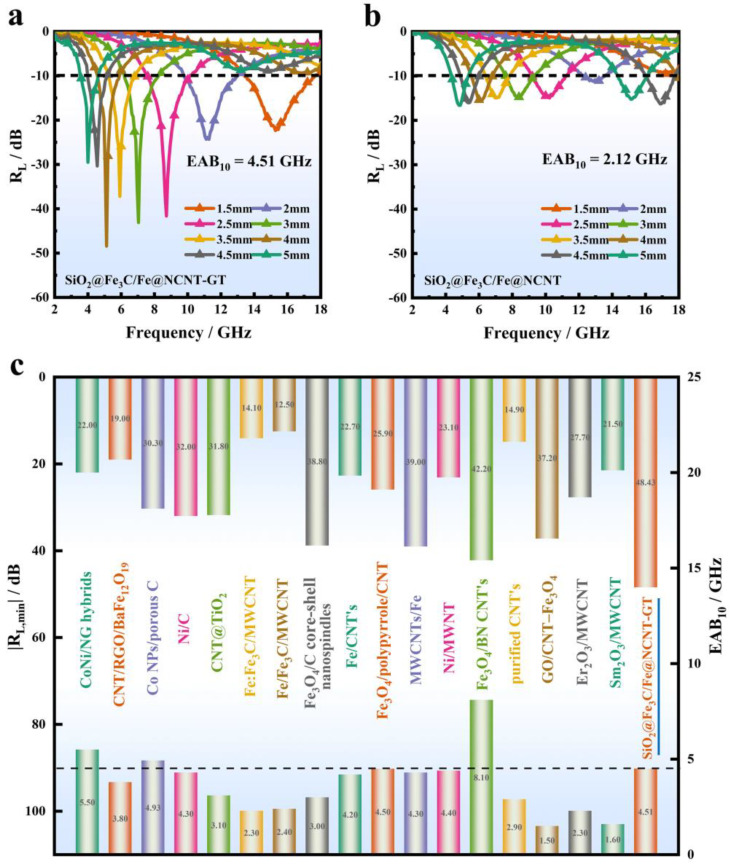
*R*_L_—*f* curves of (**a**) SiO_2_@Fe_3_C/Fe@NCNT-GT and (**b**) SiO_2_@Fe_3_C/Fe@NCNT, (**c**) the absorption performance of SiO_2_@Fe_3_C/Fe@NCNT-GT with previously reported absorbers.

In general, the dielectric loss of the absorbing material includes the conduction loss and the polarization relaxation loss within the range of gigahertz. The former can be expressed by the formula (*ε*_c_*″* = *σ/ε*_0_*ω*); the characters *σ*, *ε*_0_ and *ω* represent the conductivity, the permittivity in a vacuum and the circular frequency, respectively. The latter is expressed by the formula (*ε*_p_*″* =*ε″* − *ε*_c_*″*). The experimental results showed that the electrical conductivity of the SiO_2_@Fe_3_C/Fe@NCNT-GT was higher than that of the SiO_2_@Fe_3_C/Fe@NCNT (Table S1). Therefore, the SiO_2_@Fe_3_C/Fe@NCNT-GT had increased conductive loss compared to the SiO_2_@Fe_3_C/Fe@NCNT (Figure 4d). Meanwhile, the SiO_2_@Fe_3_C/Fe@NCNT-GT also had improved polarization losses compared to the SiO_2_@Fe_3_C/Fe@NCNT (Figure 4e) and had a higher attenuation coefficient (Figure 4f). As shown in Figure S8, multiple Cole–Cole semicircles could be found in the curve of the SiO_2_@Fe_3_C/Fe@NCNT-GT, confirming the existence of dipole polarization and interfacial polarization relaxation. Therefore, the increased dielectric relaxation loss of the SiO_2_@Fe_3_C/Fe@NCNT-GT is relevant to their enhanced dipole and interface polarizations [31,32,33,34,35]. Figure 5 shows the *R*_L_—*f* curves of the two samples with *d* of 1.5–5.0 mm over 2–18 GHz. It can be found that the SiO_2_@Fe_3_C/Fe@NCNT-GT exhibited a better EMW absorption property than the SiO_2_@Fe_3_C/Fe@NCNT. It should be noted that all of the *R*_L_ values of SiO_2_@Fe_3_C/Fe@NCNT-GT can exceed −20 dB (Figure 5a), where the minimum value was −48.43 dB with *d* of only 1.5 mm. However, the *R*_L__, m__in_ value for the SiO_2_@Fe_3_C/Fe@NCNT was only −16.63 dB with *d* of 5 mm (Figure 5b). Furthermore, the effective absorption bandwidth (EAB_10_, *R*_L_ ≤ −10 dB) of the SiO_2_@Fe_3_C/Fe@NCNT-GT was 4.51 GHz, which is superior to that of the SiO_2_@Fe_3_C/Fe@NCNT (2.12 GHz) (Figure 5a,b). Thus, the SiO_2_@Fe_3_C/Fe@NCNT-GT showed a significantly enhanced EMW absorption property in the main parameters, including *R*_L, m__in_, EAB_10_ and *d* values, showing it has potential applications in practical EMW absorption. In addition, our prepared SiO_2_@Fe_3_C/Fe@NCNT-GT had comparable, or better, EMW absorption performance than reported carbon nanotube-based absorbent materials (Figure 5c, Table S2) [36,37,38,39,40,41,42,43,44,45,46,47,48,49,50,51,52,53]. The *M*_z_—*f* plot reveals that the SiO_2_@Fe_3_C/Fe@NCNT-GT had better impedance matching characteristics compared to the SiO_2_@Fe_3_C/Fe@NCNT (Figure S9). Therefore, the increase in diameter of NCNTs may also have a positive effect on the optimization of dielectric loss and impedance matching characteristics, thus enhancing the EMW absorption performance of the SiO_2_@Fe_3_C/Fe@NCNT-GT. Overall, compared to the counterpart (SiO_2_@Fe_3_C/Fe@NCNT) without treatment in the organic solvent, the as-prepared SiO_2_@Fe_3_C/Fe@NCNT-GT showed significantly improved EMW absorption performance. In addition, SiO_2_@Fe_3_C/Fe@NCNT-GT exhibited a decreased EMW absorption performance when the filling ratio was 20% or 30% (Figure S10). Therefore, the optimal filler ratio for EMW absorption is 25%.

## 4. Conclusions

In summary, we fabricated the SiO_2_@Fe_3_C/Fe@NCNT-GT with a moderate diameter for EMW absorption. Compared to the counterpart (SiO_2_@Fe_3_C/Fe@NCNT) without treatment in the organic solvent, the dielectric loss of the as-prepared SiO_2_@Fe_3_C/Fe@NCNT-GT was optimized, the impedance matching characteristics were adjusted, and the absorption performance of EMW was significantly improved. At a filler ratio of 25%, minimum, reflection loss can reach −48.43 dB. In the meantime, effective bandwidth of the SiO_2_@Fe_3_C/Fe@NCNT-GT can reach 4.51 GHz, while the matching thickness is only 1.5 mm, which is better than most magnetic carbon matrix composites. Our present approach opens up an effective way to develop low-cost, high-performance EMW absorbers.

## Data Availability

Data is contained within the article or Supplementary Materials.

## Supplementary Materials

The following are available online at https://www.mdpi.com/article/10.3390/nano11102636/s1, Figure S1: SEM images of SiO_2_@Fe(OH)_3_ and SiO_2_@Fe(OH)_3_-GT, Figure S2: XRD patterns and FTIR spectras of SiO_2_@Fe(OH)_3_ and SiO_2_@Fe(OH)_3_-GT, Figure S3: XPS spectra of the SiO_2_@Fe_3_C/Fe@NCNT-GT, Figure S4: SEM image of SiO_2_@Fe_3_C/Fe@NCNT-GT, Figure S5: (a) TEM image, (b) Average diameter of NCNTs and NPs of SiO_2_@Fe_3_C/Fe@NCNT-GT, Figure S6: (a) *μ*′—*f* curves, (b) *μ*″—*f* curves, and (c) tan*δ*_m_—*f* of SiO_2_@Fe_3_C/Fe@NCNT-GT and SiO_2_@Fe_3_C/Fe@NCNT, Figure S7: Magnetization hysteresis loops of the SiO_2_@Fe_3_C/Fe@NCNT-GT and SiO_2_@Fe_3_C/Fe@NCNT, Figure S8: Cole-Cole semicircles of the (a) SiO_2_@Fe_3_C/Fe@NCNT-GT and (b) SiO_2_@Fe_3_C/Fe@NCNT, Figure S9: The *M*_z_—*f* curves of the (a) SiO_2_@Fe_3_C/Fe@NCNT-GT and (b) SiO_2_@Fe_3_C/Fe@NCNT, Figure S10: *R*_L_—*f* curves of (a) the SiO_2_@Fe_3_C/Fe@NCNT-GT with a filler ratio of 20 wt.% and (b) 30 wt.%, Table S1: Electrical conductivity of absorbing materials, Table S2: EMW absorption properties of some representative materials.
