# Supplementary material for: Tuning Dielectric Loss of SiO2@CNTs for Electromagnetic Wave Absorption
Source: Nanomaterials (Basel). 2021 Oct 7;11(10):2636. doi: 10.3390/nano11102636 (PMC8537562; doi:10.3390/nano11102636)
Supplement: Supplementary file 1 [file nanomaterials-11-02636-s001.zip › nanomaterials-1408388-supplementary.pdf]

*Supplementary Materials*

# Tuning Dielectric Loss of $SiO_2$@CNTs for Electromagnetic Wave Absorption

**Fenghui Cao [1,2], Jia Xu [1], Xinci Zhang [1], Bei Li [1], Xiao Zhang [1,*], Qiuyun Ouyang [1], Xitian Zhang [3] and Yujin Chen [1,4,*]**

1 Key Laboratory of In-Fiber Integrated Optics, College of Physics and Optoelectronic Engineering, Harbin Engineering University, Harbin 150001, China; caofenghui@hrbeu.edu.cn (F.C.); xujia110006@hrbeu.edu.cn (J.X.); zhangxinci@hrbeu.edu.cn (X.Z.); 1284034781@hrbeu.edu.cn (B.L.); qyouyang7823@aliyun.com (Q.O.)
2 School of Mechatronic Engineering, Daqing Normal University, Daqing 163712, China
3 Key Laboratory for Photonic and Electronic Bandgap Materials, Ministry of Education, School of Physics and Electronic Engineering, Harbin Normal University, Harbin 150025, China; xtzhangzhang@hotmail.com
4 School of Materials Science and Engineering, Zhengzhou University, Zhengzhou 450001, China
* Correspondence: zhangxiaochn@hrbeu.edu.cn (X.Z.); chenyujin@hrbeu.edu.cn (Y.C.)

## EXPERIMENTAL SECTION

### Structure Characterizations

The magnetic property of NCNTs samples were measured by a vibrating sample magnetometer (VSM; Lakeshore 7410, Columbus, USA) at room temperature.

### Electromagnetic parameter measurement

The electromagnetic wave absorption properties of the absorbing materials were measured by using a vector network analyzer (Anritsu MS4644A Vectorstar) in the 2–18 GHz range at the room temperature. The cylindrical sample (with the inner diameter and outer diameter are 3.04 mm and 7.00 mm respectively, and 3.00 mm thickness) was prepared by mixing absorbing materials with paraffin matrix was controlled to be 25 wt.% for $SiO_2$@$Fe_3C$/Fe@NCNT-GT.

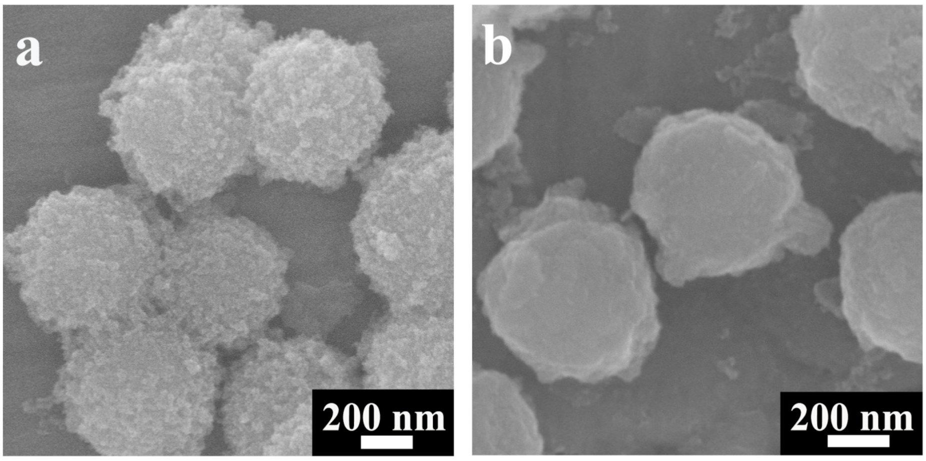


**Figure S1.** SEM images of $SiO_2$@$Fe(OH)_3$ and $SiO_2$@$Fe(OH)_3$-GT.

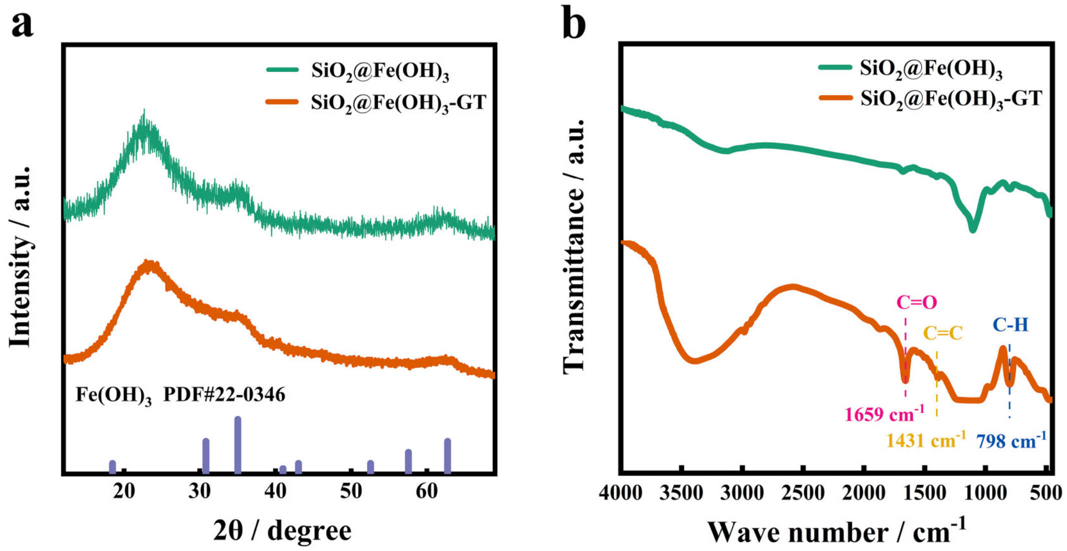


**Figure S2.** XRD patterns and FTIR spectras of $SiO_2@Fe(OH)_3$ and $SiO_2@Fe(OH)_3$-GT.

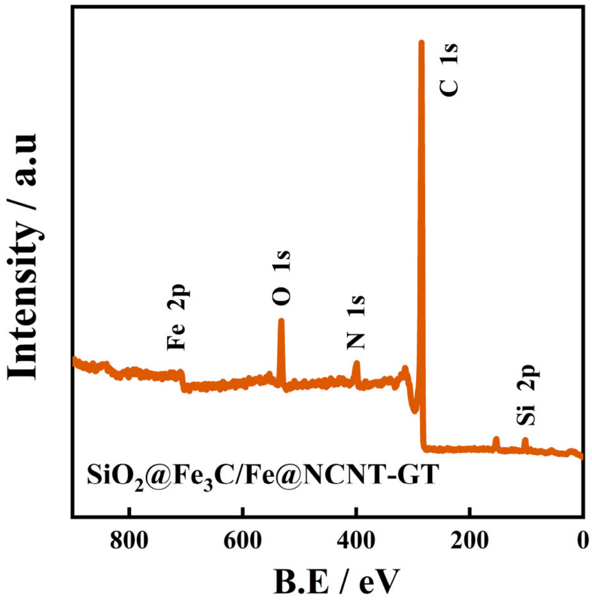


**Figure S3.** XPS spectra of the $SiO_2@Fe_3C$/Fe@NCNT-GT.

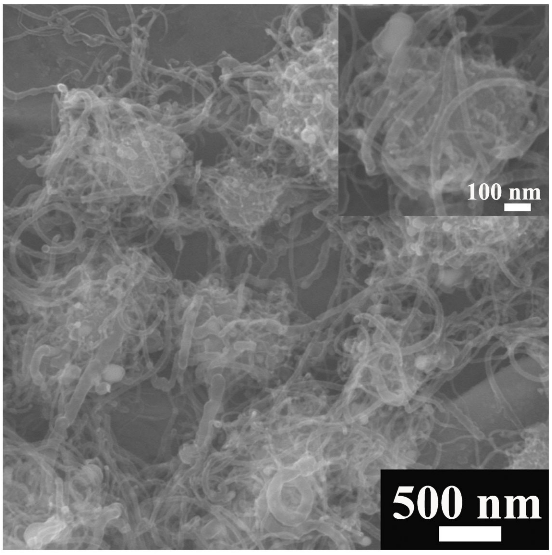


**Figure S4.** SEM image of $SiO_2@Fe_3C/Fe@NCNT-GT$.

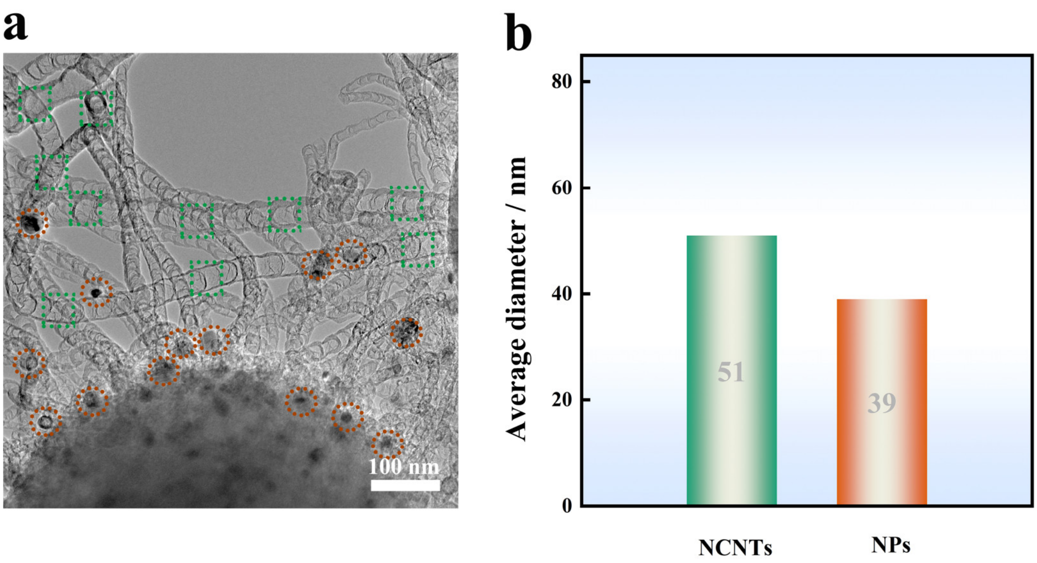


**Figure S5.** (**a**) TEM image, (**b**) Average diameter of NCNTs and NPs of $SiO_2@Fe_3C/Fe@NCNT-GT$.

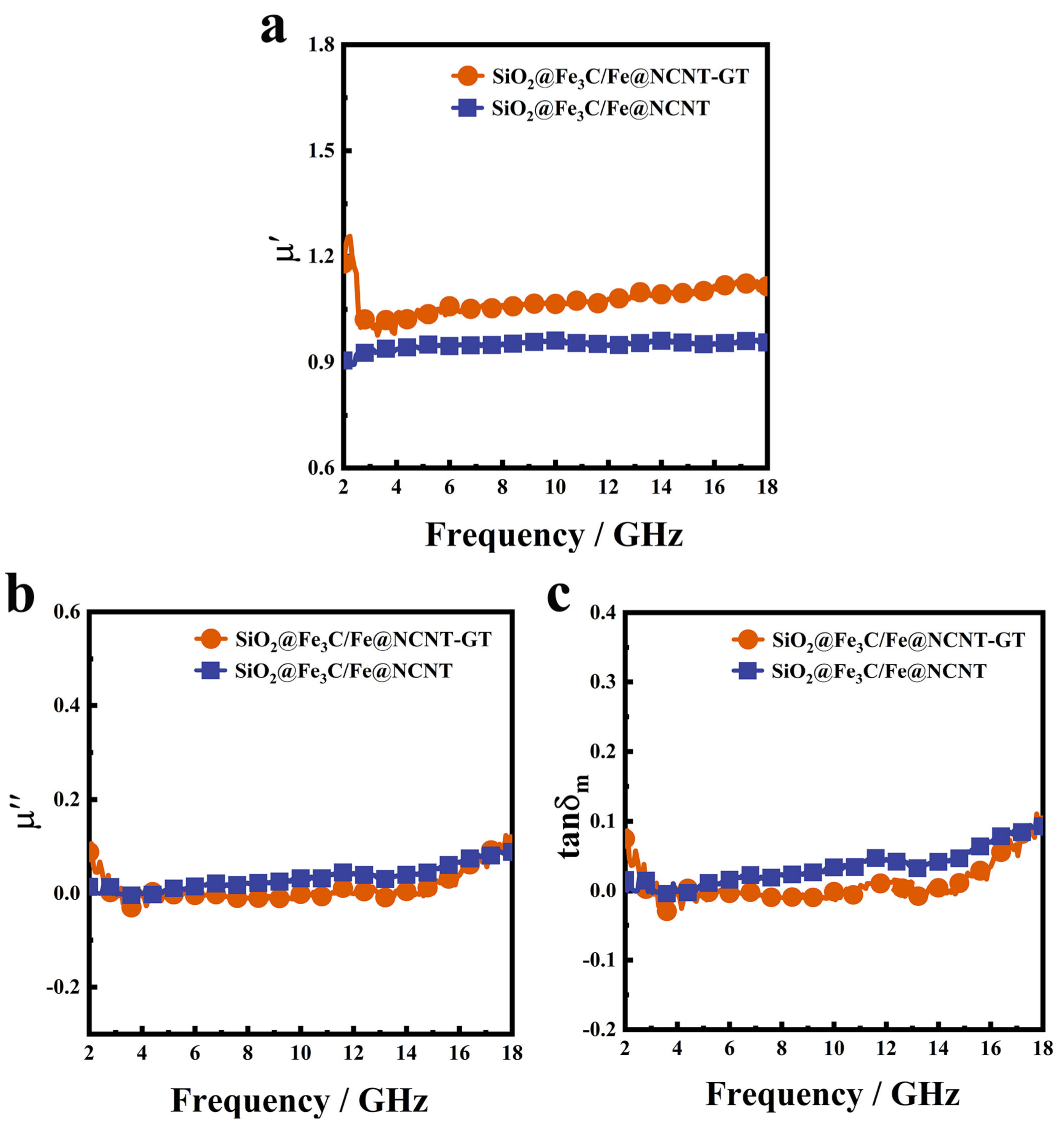


**Figure S6.** (**a**) $\mu'-f$ curves, (**b**) $\mu''-f$ curves, and (**c**) $\tan\delta_m-f$ of $SiO_2$@$Fe_3C$/Fe@NCNT-GT and $SiO_2$@$Fe_3C$/Fe@NCNT.

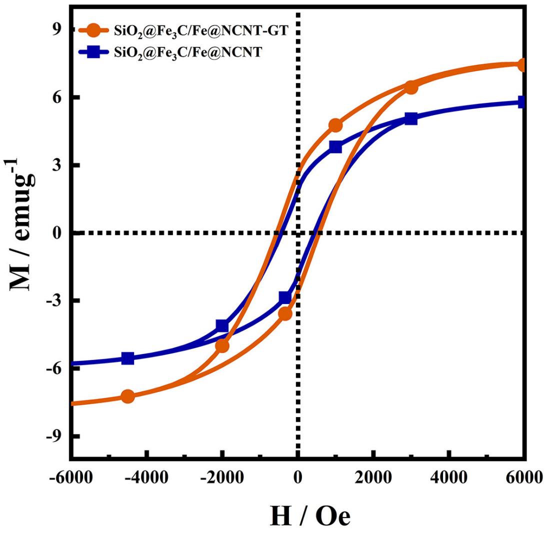


**Figure S7.** Magnetization hysteresis loops of the $SiO_2$@$Fe_3C$/Fe@NCNT-GT and $SiO_2$@$Fe_3C$/Fe@NCNT.

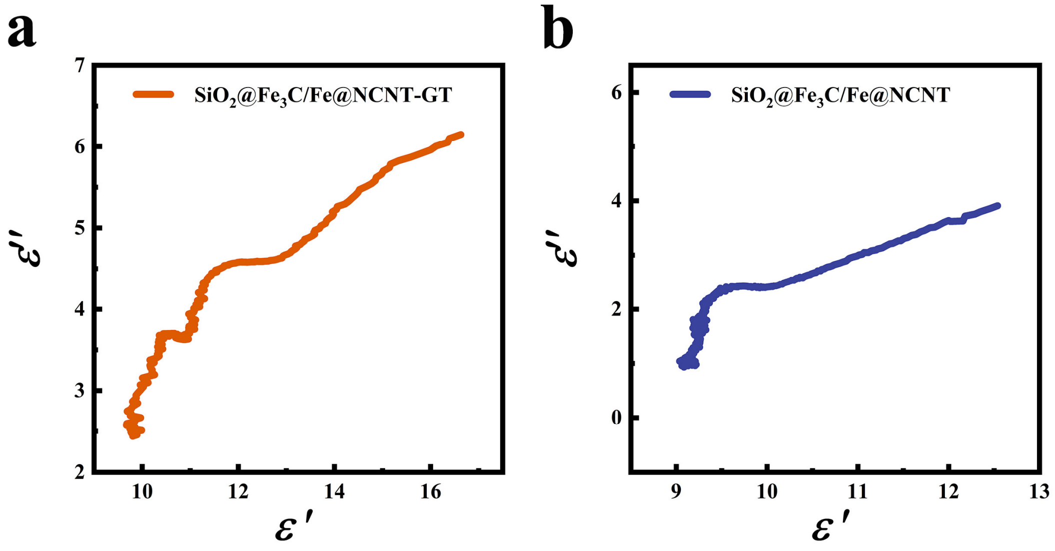


**Figure S8.** Cole-Cole semicircles of the (**a**) $SiO_2$@$Fe_3C$/Fe@NCNT-GT and (**b**) $SiO_2$@$Fe_3C$/Fe@NCNT.

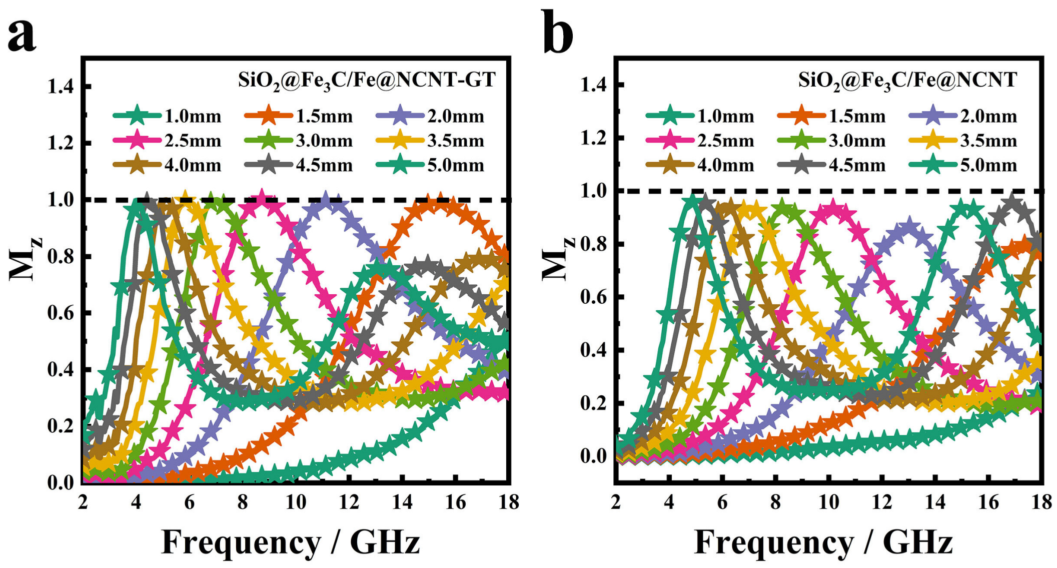


**Figure S9.** The $M_Z$—$f$ curves of the (**a**) $SiO_2$@$Fe_3C$/Fe@NCNT-GT and (**b**) $SiO_2$@$Fe_3C$/Fe@NCNT.

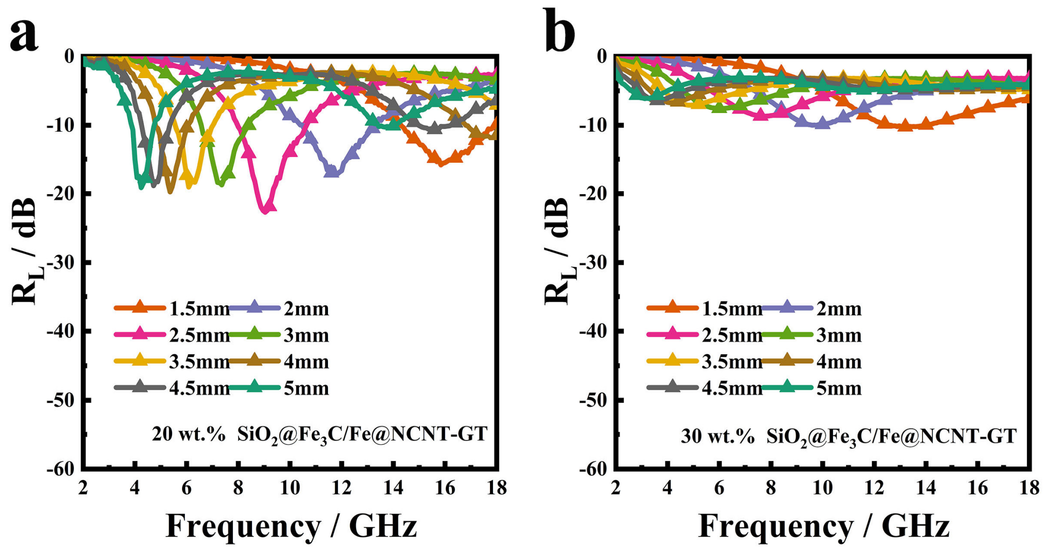


**Figure S10.** $R_L$—$f$ curves of (**a**) the $SiO_2$@$Fe_3C$/Fe@NCNT-GT with a filler ratio of 20 wt.% and (**b**) 30 wt.%.

**Table S1.** Electrical conductivity of absorbing materials.

| Sample | Electrical conductivity (S $m^{-1}$) |
| --- | --- |
| $SiO_2$@$Fe_3C$/Fe@NCNT | 0.435 |
| $SiO_2$@$Fe_3C$/Fe@NCNT-GT | 0.686 |

**Table S2.** EMW absorption properties of some representative materials.

| Sample | $f_r$ wt.% | $d$ mm | $EAB_{10}$ GHz | $R_{L,\,min}$ dB | SRL dB/m | SEAB GHz/m | Ref. |
|---|---|---|---|---|---|---|---|
| CoNi/NG hybrids | 30 | 2.0 | ~5.5 | –22.0 | 3.7 | 0.9 | [2] |
| CNT/RGO/$BaFe_{12}O_{19}$ | 20 | 2.5 | 3.8 | –19.0 | 3.8 | 0.7 | [3] |
| Co NPs/porous C | 25 | 3.0 | 4.93 | –30.3 | 4.0 | 0.6 | [4] |
| Ni/C | 50 | 2 | 4.3 | –32.0 | 3.2 | 0.4 | [5] |
| $Fe_3O_4$/C core−shell nanospindles | 60 | 2.1 | ~3.0 | –38.8 | 3.1 | 0.2 | [6] |
| CNT@$TiO_2$ | 30 | 2.0 | 3.1 | –31.8 | 5.3 | 0.5 | [7] |
| MWCNTs/Ni | 60 | ~5.2 | - | –37.0 | 1.2 | - | [8] |
| MWCNTs/Fe | 60 | ~4.3 | - | –39.0 | 1.5 | - | [8] |
| MWCNTs/Co | 60 | ~5.3 | - | –37.0 | 1.2 | - | [8] |
| $Fe_3O_4$/polypyrrole/CNT | 20 | 3.0 | 4.5 | –25.9 | 4.3 | 0.7 | [9] |
| GO/CNT–$Fe_3O_4$ | 30 | 5.0 | ~1.5 | –37.2 | 2.5 | 0.1 | [10] |
| raw CNT's | 45 | 3.0 | 0 | –9.9 | 0.7 | 0 | [10] |
| purified CNT's | 45 | 3.0 | 2.9 | –14.9 | 1.1 | 0.2 | [11] |
| Fe/CNT's | 20 | 3.5 | 4.2 | –22.7 | 3.2 | 0.6 | [12] |
| $Fe_3O_4$/BN CNT's | - | 2.0 | 8.1 | –42.2 | - | - | [13] |
| $Fe_3O_4$/BN CNT's | - | 2.5 | 13.1 | –47.9 | - | - | [13] |
| Ni/MWNT | 5 | 4.0 | 4.4 | –23.1 | 11.5 | 2.2 | [14] |
| Ni/MWNT | 10 | 4.0 | 3.4 | –17.8 | 4.4 | 0.8 | [14] |
| Fe/$Fe_3C$/MWCNT | 50 | 2.0 | 2.4 | –12.5 | 1.2 | 0.2 | [15] |
| Fe:$Fe_3C$/MWCNT | 50 | 3.5 | 2.3 | –14.1 | 0.8 | 0.1 | [15] |
| $Er_2O_3$/MWCNT | 20 | 2.0 | 2.3 | –27.7 | 6.9 | 0.6 | [16] |
| $Sm_2O_3$/MWCNT | 20 | 2.0 | 1.6 | –21.5 | 5.4 | 0.4 | [17] |
| PPy (30 wt%) | 30 | 2.6 | 6.8 | –56.3 | 7.2 | 0.9 | [18] |
| PANI | 17.5 | 2.1 | 5.5 | –25.2 | 6.8 | 1.4 | [19] |
| $SiO_2$@$Fe_3C$/Fe@NCNT-GT | 25 | 1.5 | 4.51 | –48.43 | 12.9 | 1.2 | This work |

## References


1. Stöber, W.; Fink, A.; Bohn, E. Controlled growth of monodisperse silica spheres in the micron size range. *J. Colloid Interf. Sci.* **1968** *26* 62–69.
2. Feng, J.; Pu, F. Z.; Li, Z. X.; Li, X. H.; Hu, X. Y.; Bai, J. T. Interfacial interactions and synergistic effect of CoNi nanocrystals and nitrogen-doped graphene in a composite microwave absorber. *Carbon.* **2016**, *104*, 214–225.
3. Zhao, T. K.; Ji, X. L.; Jin, W. B.; Wang, C.; Ma, W. X.; Gao, J. J.; *et al.* Direct in situ synthesis of a 3D interlinked amorphous carbon nanotube/graphene/$BaFe_{12}O_{19}$ composite and its electromagnetic wave absorbing properties. *Rsc Advances.* **2017**, *7*, 15903–15910.
4. Wang, H.C.; Xiang, L.; Wei, W.; An, J.; He, J.; Gong, C.H.; *et al.*, Efficient and lightweight electromagnetic wave absorber derived from metal organic framework-encapsulated cobalt nanoparticles. *ACS Appl. Mater. Interfaces.* **2017** *9* 42102–42110.
5. Zhang, X. F.; Dong, X. L.; Huang, H.; Liu, Y. Y.; Wang, W. N.; Zhu, X. G.; *et al.* Microwave absorption properties of the carbon-coated nickel nanocapsules. *Appl Phys Lett.* **2006**, *89*, 053115.
6. Liu, X. F.; Cui, X. R.; Chen, Y. X.; Zhang, X. J.; Yu, R. H.; Wang, G. S.; *et al.* Modulation of electromagnetic wave absorption by carbon shell thickness in carbon encapsulated magnetite nanospindles-poly(vinylidene fluoride) composites. *Carbon.* **2015**, *95*, 870–878.
7. Mo, Z.C.; Yang, R.L.; Lu, D.W.; Yang, L.L.; Hu, Q.M.; Li, H.B. *et al.*, Lightweight, three-dimensional carbon nanotube@$TiO_2$ sponge with enhanced microwave absorption performance. *Carbon.* **2019,** *144*, 433–439.
8. Wen, F. S.; Zhang, F.; Liu, Z. Y. Investigation on microwave absorption properties for multiwalled carbon nanotubes/Fe/Co/Ni nanopowders as lightweight absorbers. *J Phys Chem C.* **2011**, *115*, 14025–14030.
9. Zhou, Y.; Miao, J.; Shen, Y.; Xie, A. Novel porous $Fe_xC_yN_z$/N-doped CNT nanocomposites with excellent bifunctions for catalyzing oxygen reduction reaction and absorbing electromagnetic wave. *Applied Surface Science.* **2018**, *453*, 83–92.
10. Wang, L. N.; Jia, X. L.; Li, Y. F.; Yang, F.; Zhang, L. Q.; Liu, L. P.; *et al.* Synthesis and microwave absorption property of flexible magnetic film based on graphene oxide/carbon nanotubes and $Fe_3O_4$ nanoparticles. *Journal Of Materials Chemistry A.* **2014**, *2*, 14940–14946.
11. Qi, X.; Xu, J.; Hu, Q.; Deng, Y.; Xie, R.; Jiang, Y.; *et al.* Metal-free carbon nanotubes: synthesis, and enhanced intrinsic microwave absorption properties. *Scientific reports.* **2016**, *6*, 28310.
12. Lin, H.; Zhu, H.; Guo, H.; Yu, L. Investigation of the microwave-absorbing properties of Fe-filled carbon nanotubes. *Materials Letters.* **2007**, *61*, 3547–3550.
13. Zhang, T.; Zhong, B.; Yang, J. Q.; Huang, X. X.; Wen, G. Boron and nitrogen doped carbon nanotubes/$Fe_3O_4$ composite architectures with microwave absorption property. *Ceramics International.* **2015**, *41*, 8163–8170.

14. Zou, T.; Li, H.; Zhao, N.; Shi, C. Electromagnetic and microwave absorbing properties of multi-walled carbon nanotubes filled with Ni nanowire. *Journal of Alloys and Compounds.* **2010**, *496*, L22–L4.
15. Xu, P.; Han, X. J.; Liu, X. R.; Zhang, B.; Wang, C.; Wang, X. H. A study of the magnetic and electromagnetic properties of γ-$Fe_2O_3$–multiwalled carbon nanotubes (MWCNT) and Fe/$Fe_3C$–MWCNT composites. *Materials Chemistry and Physics.* **2009**, *114*, 556–560.
16. Zhang, L.; Zhu, H.; Song, Y.; Zhang, Y.; Huang, Y. The electromagnetic characteristics and absorbing properties of multi-walled carbon nanotubes filled with $Er_2O_3$ nanoparticles as microwave absorbers. *Materials Science and Engineering: B.* **2008**, *153*, 78–82.
17. Zhang, L.; Zhu, H. Dielectric, magnetic, and microwave absorbing properties of multi-walled carbon nanotubes filled with $Sm_2O_3$ nanoparticles. *Materials Letters.* **2009**, *63*, 272–274.
18. Green, M.; Tran, A. T. V.; Chen, X. Maximizing the microwave absorption performance of polypyrrole by data-driven discovery. *Composites Science and Technology.* **2020**, *199*, 108332..
19. Green, M.; Tran, A. T.; Chen, X. Obtaining Strong, Broadband microwave absorption of polyaniline through data-driven materials discovery. *Advanced Materials Interfaces.* **2020**, *7*, 2000658.
